# Supplementary material for: C-type natriuretic peptide improves maternally aged oocytes quality by inhibiting excessive PINK1/Parkin-mediated mitophagy
Source: eLife. 2023 Oct 20;12:RP88523. doi: 10.7554/eLife.88523 (PMC10588981; doi:10.7554/eLife.88523)
Supplement: Supplementary file 1. [file elife-88523-supp1.docx]

**Table S1 Primers sequences**

| Gene name | Primer sequences | Amplicon size (bp) | NCBI Reference Sequence | |
| --- | --- | --- | --- | --- |
| *Gapdh* | F: 5’-TCACTGCCACCCAGAAGA-3’  R: 5’-GACGGACACATTGGGGGTAG-3’ | 185 | | XM_017321385.2 |
| *Zfp640* | F: 5’-TGTGCAGGCTTGAATGGTTC-3’  R: 5’-CTGAGGTCCCCTCAATGCAC-3’ | 70 | | XM_030247511.2 |
| *Gm6749* | F: 5’-ATGGGTGTTGCCCATACCAC-3’  R: 5’-GGCTTTTGTGGCCAGTTGTT-3’ | 93 | | XM_017321875.3 |
| *Obox7* | F: 5’-TTGTCCGCAAGAATACCAAGAA-3’  R: 5’-AGAGCTTGTCTGCAGATGGAC-3’ | 188 | | NM_001038676.1 |
| *Glrx* | F: 5’-CATAGGCGGATGCAGTGATCT-3’  R: 5’-CTCTGCCTGCCACCCCTTTTAT-3’ | 106 | | NM_053108.4 |
| *Vamp9* | F: 5’-ACTCTCTTTATTGACGGAATCACT-3’  R: 5’-TATTCCATCATGTGCTGTGCT-3’ | 194 | | NM_001378420.1 |
| *Tnk2* | F: 5’-CAAGAGGGCCAGGTAGTGTG-3’  R: 5’-TTGGCACTAGAGCAACCCTG-3’ | 170 | | NM_016788.3 |
| *Cd72* | F: 5’-CCTCGGAAGTCTGGAGGAGA-3’  R: 5’-GGGGCGTCAGAGAGGTATTC-3’ | 108 | | NM_001110320.1 |
